# Rhombic-Shaped Channel Stent with Enhanced Drug Capacity and Fatigue Life

**DOI:** 10.3390/mi9010003

**Published:** 2017-12-24

**Authors:** Hao-Ming Hsiao, Cheng-Han Lin, Yung-Kang Shen, Tzu-Yun Chou, Yen-Yu Hsu

**Affiliations:** 1 Department of Mechanical Engineering, National Taiwan University, Taipei 10617, Taiwan; r01522830@ntu.edu.tw (C.-H.L.); r05522822@ntu.edu.tw (T.-Y.C.); b03502042@ntu.edu.tw (Y.-Y.H.); 2 Research Center for Biomedical Devices, Taipei Medical University, Taipei 11031, Taiwan; ykshen@tmu.edu.tw

**Keywords:** drug-eluting stent, rhombic-shaped drug reservoir, programmable drug delivery, channel stent, depot stent

## Abstract

A drug-eluting stent with rhombic-shaped drug reservoirs is proposed, aimed at providing long-term drug delivery and enhanced fatigue life. Unique rhombic-shaped reservoirs or channels on the stent struts can increase the total drug capacity and improve the stress distribution for longer fatigue life, without compromising other important clinical attributes. Our rhombic-shaped channel stent increases the total drug capacity by multiple times. Its fatigue safety factor, even with the large rhombic cutouts on the stent struts, could be 50% higher than that of the conventional drug-eluting stent. A pulsed fiber-optic laser and a series of expansions and heat treatments were used to make the first prototype of our rhombic-shaped channel stent. This new concept may open up a wide variety of new treatment options and opportunities for the medical industry in the future.

## 1. Introduction

A stent is a tiny, coiled wire-mesh tube that can be deployed into an artery and expanded percutaneously with a catheter during angioplasty. It is designed to open a narrowed artery that has become clogged by the buildup of cholesterol or fatty substances over time. For over two decades, the bare metal stent (BMS) was a major treatment option for coronary artery stenosis; however, a common occurrence in earlier days was in-stent restenosis, the re-narrowing of the artery after the stenting procedure [1,2,3]. Due to artery wall injury during stenting, intimal cells could proliferate, often leading to excessive neo-intimal hyperplasia and restenosis.

Since 2003, stent technology has evolved from the bare metal stent to the drug-eluting stent (DES). A drug-eluting stent is a bare metal stent coated with a drug (e.g., Sirolimus and its analogues, Paclitaxel) that is known to intervene in the cell cycle and thus the restenosis process. Sirolimus and Paclitaxel inhibit the cell cycle via different mechanisms. Sirolimus has a cytostatic effect and induces cell cycle arrest in the late G1 phase, which has been shown to inhibit all phases of the restenosis cascade, inflammation, neointimal hyperplasia formation, and smooth muscle cell migration [4,5,6]. Its analogues include Everolimus for the Xience stent (Abbott) [7], Zotarolimus for the Endeavor stent (Medtronic) [8], Biolimus A9 for the BioMatrix stent (Biosensors) [9], Tacrolimus for the Janus stent (Bioimplant S.A.) [10], etc. Paclitaxel acts on the G2/M phase of the cell cycle, but cell killing is dependent on drug concentrations and duration of the cell exposure [6,11,12]. It was used in Boston Scientific’s earlier stents such as the Express stent and the Liberté stent.

The drug is typically mixed with a polymer compound, whether durable or biodegradable, to precisely control the release rate of the drug to the artery wall. However, biocompatibility and other issues are often associated with these polymer carrier systems. An alternate approach is the use of polymer-free drug-eluting stents, yet preventing functionalities of the polymer carrier [13,14,15]. The birth of the drug-eluting stent has dramatically reduced restenosis rates from 20% to 30% for the bare metal stent to less than 10% now, leading to worldwide application of this technology in the medical industry. The success of the drug-eluting stent has made it the gold standard for the treatment of coronary artery stenosis. However, in attempts to further perfect the percutaneous management of intravascular diseases, numerous innovations have emerged over the last decade, and the medical industry continues to seek an ultimate solution. One of the intriguing concepts is the depot or channel stent: a drug-eluting stent laser-drilled with tiny holes or channels, called “reservoirs”, that can be loaded with one or multiple drugs for smart/programmable drug delivery, potentially in various doses or formulations with different drug-release time frames [16,17].

**Figure 1:**
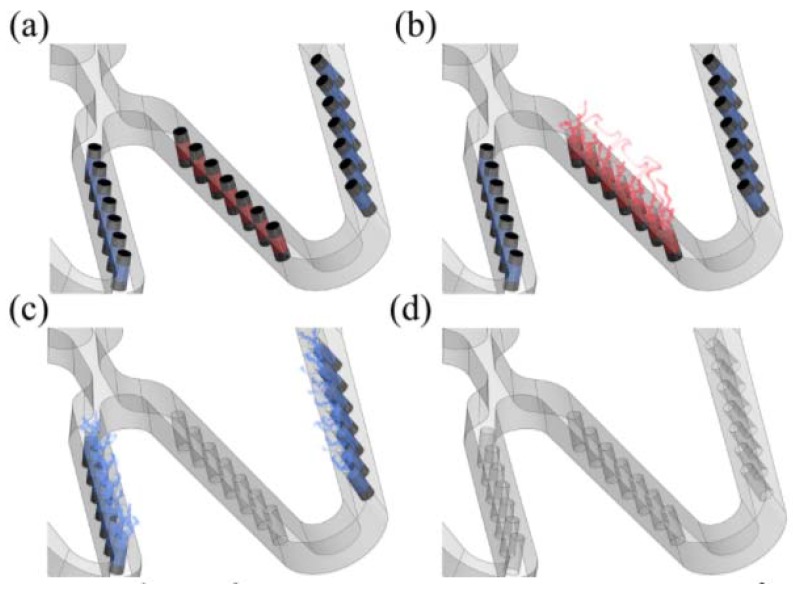
Schematic of sequential release of multiple drugs for each period after intervention: (**a**) depot stent with tiny reservoirs loaded with drugs A and B; (**b**) drug A eluted first; (**c**) drug B eluted next; (**d**) both drugs A and B completely eluted.

Unlike typical drug-eluting stents, the depot or channel stent does not require a surface coating. Its drug release can be controlled by filling reservoirs with various drugs and coatings. The drug-polymer mix can be altered from one reservoir to the next, allowing the highly controlled release of different medications (Figure 1). Furthermore, this depot or channel stent could become more powerful in some cases, such as in renal applications for potential dual treatments of renal artery stenosis (RAS) and its associated kidney diseases simultaneously. For example, drugs preventing neo-intima proliferation can be loaded on the outside portion of the reservoirs, while drugs for kidney diseases can be loaded on the inside portion to be carried by the bloodstream to the downstream kidney for target therapy. This proposed concept could potentially treat two symptoms at once.

Few depot or channel stents are available on the market today. The NEVO sirolimus-eluting stent (Cordis, Bridgewater Township, NJ, USA), the first depot stent launched, is distinct from all other drug-eluting stents due to its uniformly spaced reservoirs for containing the drug-polymer compound instead of a conformal polymer coating overlaid on the stent surface. The Firehawk rapamycin-eluting stent (MicroPort Medical, Shanghai, China) and the Janus tacrolimus-eluting stent (Sorin Biomedica, Saluggia, Italy) are channel stent variations designed with constant-width channels or grooves that serve as reservoirs on the stents. The Firehawk stent is claimed to have the world’s lowest drug dosage, with only a fraction of the drug loadings of other drug-eluting stents. The Janus stent is unique in that, once the drug tacrolimus is loaded into the reservoirs, the stent is then coated with a surface layer that prevents stent thrombosis and enhances biocompatibility [18].

The depot or channel stent has other potential advantages. In addition to the programmable drug release feature mentioned above, the depot or channel stent is free of surface coating layers, thereby reducing the contact between the artery wall and the polymer compound, which is believed to pose the risk of chronic inflammation or stent thrombosis [19,20,21]. Another advantage is the reduction of the overall stent profile due to the absence of surface coating layers. This advantage allows physicians easier access to smaller lesion sites with greater delivery control [22,23]. Despite these potential advantages, however, the drug reservoirs may weaken the stent structure and compromise its mechanical integrity, namely, its ability to sustain various loading conditions such as crimping inside a catheter, stent expansion, radial resistance to blood vessels from collapsing, and fatigue resistance to pulsatile systolic/diastolic pressures. Therefore, in this paper, a new channel stent design concept with rhombic-shaped drug reservoirs is proposed; not only to increase the total drug capacity but also to enhance the fatigue life of the stent, without trading off other major clinical attributes. This new stent concept is intended for the treatment of peripheral artery diseases and has the great potential to be extended to other diseases. For example, its high drug capacity could transform a vascular stent into a drug carrier for longer-term therapies such as cancer treatment. Our goal is to design a stent that not only offers physical support to the blood vessel but also presents a pharmacological approach for the treatment of other diseases. The stent, acting as a drug carrier in this scenario, could be deployed in an artery upstream of the distal cancer site, with cancer drugs loaded in the reservoirs to be carried by the bloodstream to the downstream cancer cells for targeted therapy. The new stent concept proposed in this paper is the extension of the previous work conducted by the authors, with more systematic approaches and affirmative conclusions [24].

Nitinol (NiTi) is a remarkable material that opens up a wide variety of applications for many industries. Some of its major uses are in medical devices where its unique super-elastic properties allow minimally invasive surgery and implants to improve quality of life for millions of people. Among them, the self-expanding stent is the most celebrated application of nitinol in medical devices [25,26,27,28]. Nitinol stent technologies continue to undergo significant improvement with a goal toward increased durability and conformability in the peripheral arteries with better long-term patency. Unlike the balloon-expandable stent which is limited to a human’s rib cage and some abdominal areas, the self-expanding stent can be implanted in any part of the body due to its crush-resistant properties. It has been used to treat peripheral artery diseases in the aorta, carotid, renal, iliac, superficial femoral, popliteal, and tibial arteries [29,30,31]. Nitinol’s versatility has been even extended to other diseases such as heart valve, biliary duct, and ventricular septal defect occluder, just to name a few [32,33,34]. With the increasing numbers of people longing for life quality, the availability of advanced materials such as nitinol is essential to allow continued progress in improving lives across the world. Since our goal is to design a stent which could also be used as a drug carrier, we believe nitinol is the best material candidate for such challenging tasks.

In recent years, computational modelling has emerged as an important tool for optimization of stent designs, and it can be used along with experimental data to improve stent performance. Such computational models could give insights into various aspects of stent design that may consequently reduce the clinical risk. Furthermore, computational modelling also provides extensive information under highly controlled conditions, making it feasible to screen various stent design iterations prior to costly prototyping. Therefore, in this paper, several computational models were developed by using finite element analysis (FEA) to evaluate stent mechanical integrity and fatigue resistance to various loading conditions for our channel stent. A pulsed fiber-optic laser and a series of expansions and heat treatments were used to make the first prototype of our rhombic-shaped channel stent.

## 2. Materials and Methods

### 2.1. Channel Stent with Rhombic-Shaped Drug Reservoirs

**Figure 2:**
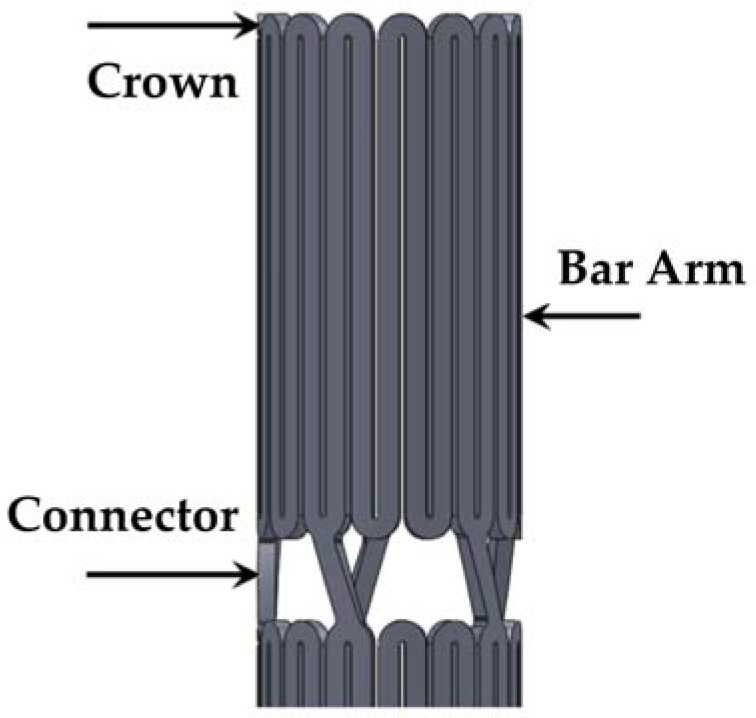
Definitions of three most important components of an intravascular stent.

A stent is a mechanical assembly of a series of nested rings interconnected with bridging connectors. Figure 2 illustrates an intravascular stent and the definitions of its three most important components (crown, bar arm, and connector).

**Figure 3:**
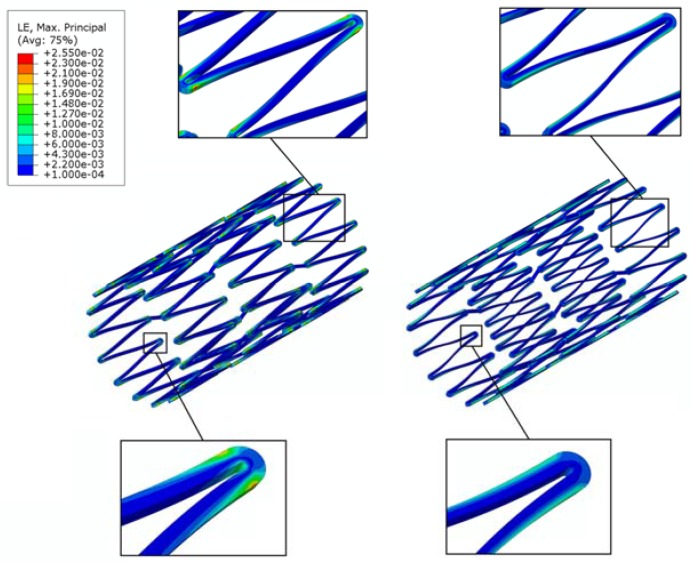
Finite element analysis (FEA) contour plot comparison of strain distribution between conventional stent (**left**) and tapered-strut stent (**right**).

It is well-known that during loading, the maximum stresses/strains occur at the most highly curved region of a stent, called the “crown”, whereas there are little to no stresses/strains on the straight portion of a stent, called the “bar arm”, which connects the two crowns. Direct modification on the stress-free bar arms has the potential to alleviate the stress concentration issue on the crown, with minimal negative impact on the overall stent behavior. In the previous studies conducted by the authors, it was shown that the fatigue safety factor could be increased by multiple times when the bar arms were tapered [35,36]. This tapered-strut design concept shifts the highly concentrated stresses/strains away from the crown, thus allowing the stress-free bar arm to carry more loading by narrowing the strut width at its mid-section to various degrees. Figure 3 shows the FEA contour plot comparison of the strain distribution between the conventional stent and the tapered-strut stent. It is evident that the strain distribution is significantly improved by such a simple design concept.

In the conventional channel stent, the width of the cutout remains constant along the entire length of the reservoirs. The creation of these reservoirs may weaken the stent structure and thus compromise its mechanical integrity and fatigue life. Following the aforementioned tapered-strut concept, instead of tapering the bar arms, a novel channel stent design with rhombic-shaped drug reservoirs is proposed. The unique rhombic shape of the reservoirs draws the stresses/strains away from the crowns and provides a smoother transition of stresses/strains along the bar arms. It was hoped that such a design could achieve similar effects of stress/strain redistribution and thus enhanced the fatigue life of the stent (the stent fatigue life).

**Figure 4:**
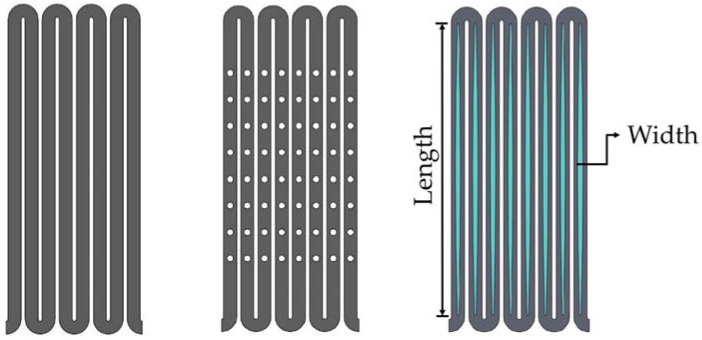
Conventional drug-eluting stent (**left**), conventional depot stent (**middle**), and our rhombic-shaped channel stent (**right**).

Figure 4 shows 2-D sketches of the three different stent designs used for comparison in this study: a conventional drug-eluting stent, a conventional depot stent, and our rhombic-shaped channel stent. The conventional drug-eluting stent is coated with one drug on its surface, whereas the conventional depot stent is laser-cut with tiny holes loaded with one or multiple drugs for drug delivery. Depending on the stent design, the diameter of the depot reservoirs varies in size and the depth is either a blind-hole or through-hole configuration. In this study, the depot stent used for comparison had a reservoir diameter of 50% of the strut width and a depth of 100% of the strut thickness. Each stent strut had eight reservoirs, and the spacing between two neighboring reservoirs, defined by the distance between two reservoir centers along the strut centerline, was 0.33 mm. For our rhombic-shaped channel stent, the effects of the reservoir size, namely, the reservoir length and width, on the stent mechanical integrity were investigated. To sketch such a stent, a rhombic-shaped cutout was first made, starting from the strut centerline tangent to the crown arc and then gradually widening towards the midpoint of the bar arm, the center of the reservoir. This reservoir length was equal to the entire length of the bar arm and was thus defined as “100% strut length” for the subsequent analysis, as represented by the blue color in Figure 4.

### 2.2. Materials

Nitinol (NiTi) is an excellent material for medical devices due to its unique super-elastic and shape-memory properties. Due to the transformation between the austenite and martensite phases, nitinol is able to return to its original shape after the loading is removed or the temperature is changed. The self-expanding stent is the most celebrated application of nitinol in the medical industry. It has been used to treat peripheral artery diseases in the aorta, carotid, renal, iliac, superficial femoral, popliteal, and tibial arteries. Due to the complex articulation of these peripheral arteries, self-expanding stents are required to be flexible, yet durable and crush-resistant. However, a fracture rate of up to 50% has been reported in some cases, raising concerns about stent fractures in peripheral artery stenting. These stent fractures are likely due to the in vivo repetitive deformations of vascular motion, and thus stent fatigue life is a major issue in these applications.

The making of a self-expanding stent requires an alternating series of expansion and heat treatment procedures as it is forged into its final shape and dimensions. The repeated heat treatment in each incremental expansion relieves the residual stresses within a stent, thus allowing it to be shaped to a larger size without stent fractures. The super-elasticity allows the stent to self-expand to its target shape and dimensions once the constraint force is removed from the stent.

### 2.3. Finite Element Models

Computational modeling has emerged as an important tool for optimization of stent designs. It is able to provide insights into various aspects of the stent behavior, which may consequently improve its clinical outcome [37,38,39,40,41,42,43]. Several finite element models were developed to evaluate the mechanical integrity and pulsatile fatigue resistance of a stent subjected to various loading conditions consistent with the current practice in the industry. These include multiple stent expansions and their corresponding heat treatments during manufacturing, crimping of the stent inside a catheter and its targeted release into a blood vessel, and pulsatile fatigue resistance under the systolic/diastolic pressure cycles of cardiac rhythm. These procedures were simulated by the following steps:

Step 1: Expand the stent to 4.0 mm, 6.0 mm, 8.0 mm, 10.0 mm Inner Diameter (ID) and heat treat the stent after each expansion for stress relief.
Step 2: Crimp the stent inside the 2.0 mm ID catheter delivery system.
Step 3: Release the stent into the 7.0 mm ID blood vessel.
Step 4: Simulate stent pulsatile fatigue resistance under systolic/diastolic pressure cycles by applying ±3% stent diameter oscillation.

Finite element analysis was performed using the ABAQUS/standard finite element solver (Dassault Systems Simulia Corp., Providence, RI, USA) with the user-defined super-elastic material subroutine user material subroutine (UMAT) [44,45]. Two cylinders with diameters of 1 mm and 11 mm were added to the model, one inside the stent and the other outside the stent. During each expansion and its subsequent heat treatment, the inner cylinder was expanded to the target diameter, and the resulting stress/strain values in all elements of the model were reset to zero to simulate the stress relief during heat treatment. The stent deformation geometry was then extracted for the next expansion until the final stent diameter was reached. After completion of the final expansion, the outer cylinder was used to crimp the stent to the catheter size for delivery. A third cylinder of 7 mm was then used to imitate the inner wall of the blood vessel. When the catheter was removed, the stent sprang back toward its final expansion size until it hit the 7 mm cylindrical surface and came to a stop.

The stent and cylinders were meshed with the eight-node linear brick element (C3D8). A frictionless contact was defined to prevent surface penetration with the following contact pairs considered: (1) surface contact between the inner surface of the outer cylinder and the outer surface of the stent, and (2) surface contact between the outer surface of the inner cylinder and the inner surface of the stent. The nitinol material behavior for the ABAQUS UMAT subroutine inputs was obtained from Pham et al. [46]. The material ultimate strain and fatigue endurance limits used in the fatigue life analysis were 10% and 1%, respectively [47,48].

### 2.4. Fatigue Life Analysis

Due to the repetitive deformations of vascular motion, a major issue in peripheral artery stenting is stent fatigue life. Goodman fatigue life analysis has been widely used in the medical device industry for assessing fatigue resistance and providing an indication of the chronic durability of a device. It is also recommended by the Food and Drug Administration (FDA) that Goodman fatigue life analysis be used to determine the stent fatigue safety factor under physiologic loading of up to 4 × 10^8^ fatigue cycles. After simulations (Steps 1–3) on stent manufacturing and deployment, a ±3% stent diameter oscillation was applied to simulate the pulsatile motion of the blood vessel. For the self-expanding stent, modified strain-based Goodman fatigue life analysis was used due to the unique properties of nitinol [27,28,44,49,50,51]. According to that analysis, fatigue failure occurs if the strain state within a stent satisfies the following relation:

(1) $$\left(\frac{\varepsilon_{a}}{\varepsilon_{e}}\right)+\left(\frac{\varepsilon_{m}}{\varepsilon_{u}}\right)\geq 1$$

where *ε_a_* is the strain amplitude applied to the device, *ε_e_* is the material endurance limit, *ε_m_* is the mean strain applied to the device, and *ε_u_* is the material ultimate strain. The Goodman diagram is a plot of the normalized strain amplitude *ε_a_*/*ε_e_* (*y*-axis) versus the normalized mean strain *ε_m_*/*ε_u_* (*x*-axis). The equation *ε_a_*/*ε_e_* + *ε_m_*/*ε_u_* = 1 represents the stent failure line.

The fatigue safety factor (*FSF*) is defined as the ratio of the strain amplitude against the endurance limit. An *FSF* of less than 1.0 indicates stent fatigue failure.

(2) $$FSF=\frac{\varepsilon_{e}}{\varepsilon_{a}}$$

### 2.5. Channel Stent Prototyping

**Figure 5:**
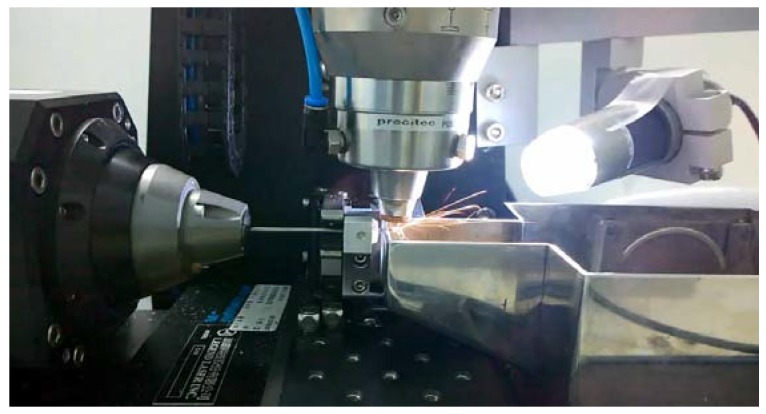
Cutting the design pattern of the rhombic-shaped channel stent onto a hypotube by laser.

A laser module consisting of a Rofin (Rofin-Baasel Lasertech GmbH & Co., Gilching, Germany) 100 W pulsed fiber laser, an Aerotech (Aerotech, Inc., Pittsburgh, PA, USA) linear *X*-*Y* motor stage, and a *Z*-direction server motor were assembled and integrated. During the manufacture of the rhombic-shaped channel stent, a 2-D drawing was first sketched on the *X*-*Y* plane and then coded into the 3-D cylindrical coordinate system by wrapping the 2-D sketch around a cylinder of the target size. The device design pattern was cut onto a seamless 2.0 mm hypotube based on the coded geometry uploaded to the laser module. The linear motion and rotation of the hypotube were provided by the linear *X*-*Y* motor stage, while the distance between the laser source and hypotube surface was controlled by the *Z*-direction server motor for optimal focal position. Position Synchronized Output (PSO) was the control algorithm used to coordinate the linear *X*-*Y* motor stage with the timing of laser firing. This algorithm greatly enhanced the laser-cutting quality and laser output efficiency (Figure 5).

## 3. Results

### 3.1. Effectiveness of Stress/Strain Redistribution

**Figure 6:**
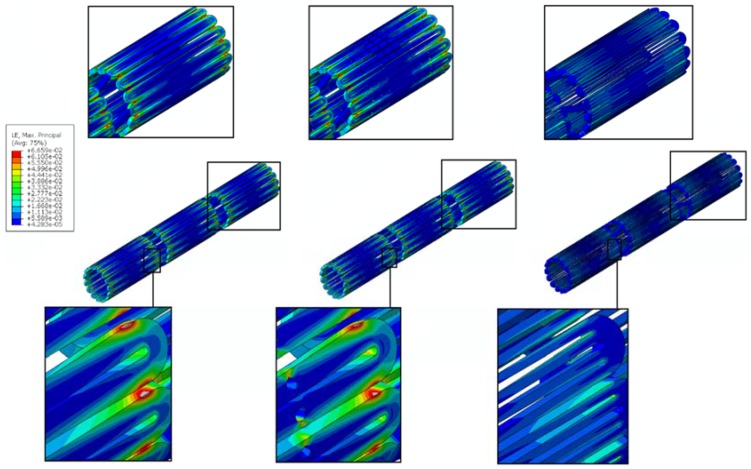
FEA contour plot comparison of strain distribution at 2.0 mm after crimping into a catheter among the conventional drug-eluting stent (DES) (**left**), conventional depot stent (**middle**), and rhombic-shaped channel stent (**right**).

**Figure 7:**
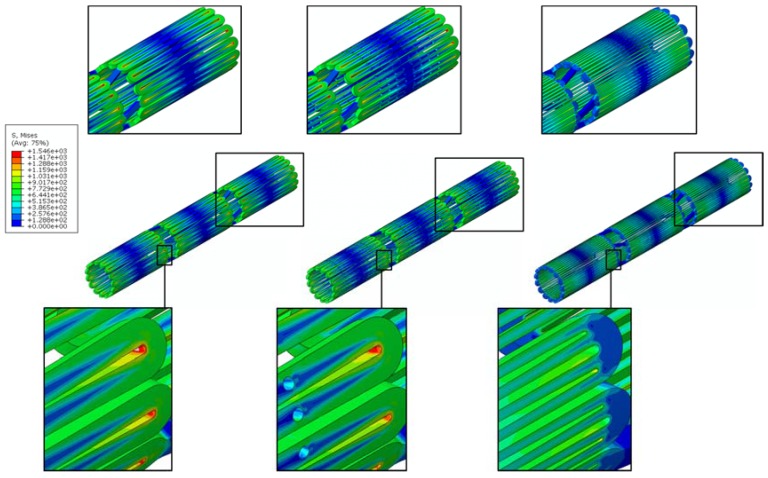
FEA contour plot comparison of von Mises stress distribution at 2.0 mm after crimping into a catheter among the conventional DES stent (**left**), conventional depot stent (**middle**), and rhombic-shaped channel stent (**right**).

A channel stent design with rhombic-shaped drug reservoirs is proposed, based on the tapered-strut concept for enhanced drug capacity and fatigue life. Figure 6 and Figure 7 show the contour plot comparison of the strain distribution and von Mises stress distribution, respectively, for the three different stent types after crimping into a 2 mm catheter. In this specific comparison, the channel stent had a reservoir size of 100% of the strut length and 40% of the strut width at the midpoint of the bar arm. Crimping is the most critical step of all the procedures, as the highest strain occurs during this stage of manufacturing and deployment. Severe stress concentrations occurred near the crown regions of the conventional DES stent and thus presented an issue, as indicated by the red color. The same phenomenon was also observed for the conventional depot stent, not only at the crown regions but also around the reservoirs. In contrast, the stress concentration was significantly alleviated in our rhombic-shaped channel stent. Due to the unique rhombic shape of the reservoirs, the energy was redistributed on the stent struts more uniformly and efficiently, successfully drawing the highly concentrated stresses/strains away from the crown regions and towards the stress-free bar arms.

### 3.2. Effects of Reservoir Length

To investigate the effects of the rhombic-shaped reservoir on drug capacity and fatigue resistance, the reservoir length was varied from 100% to 40% of the strut length (SL) in 10% increments, while the reservoir width at the midpoint of the bar arm was maintained at 40% of the strut width.

#### 3.2.1. Total Drug Capacity

For drug-eluting stents, in addition to the ability to support the artery wall, one of the important functional attributes is drug release to suppress endothelial cell proliferation. Table 1 presents the comparison of the total drug capacities among the three different stent types: the conventional DES stent, the conventional depot stent, and our rhombic-shaped channel stent with various strut lengths. The conventional DES stent has a coating thickness of about 5 μm, carrying a total drug capacity equivalent to the stent surface area times its coating thickness. For the conventional depot stent presented in this paper, each bar arm had eight reservoirs, and each reservoir size was 50% of the strut width in diameter and 100% of the strut thickness in this specific design. Although the fatigue resistance of the conventional depot stent was somewhat impacted by the creation of the drug reservoirs, its total drug capacity was increased to 1.65 times that of the conventional DES stent, which is sufficient to fully replace the coating layers on the stent surface, completely eliminate the surface coating, and offset the marginal loss in fatigue life.

Table 1 also shows that total drug capacity of our rhombic-shaped channel stent was up to 7.66 times that of the conventional DES stent. When the reservoir length was decreased, the reservoir volume and thus the total drug capacity became lower. Even with the shortest reservoir length investigated (40% of the strut length), the total drug capacity was still increased by 3.07 times. This demonstrates that, in terms of the total drug capacity, the rhombic-shaped channel stent outperformed both the conventional DES stent and the conventional depot stent. However, reservoir lengths shorter than 40% of the strut length were not investigated, as the total drug capacity was only marginally greater than that of the conventional depot stent.

It should be noted that these comparison and measurements were made based on the cases where Everolimus drug was mixed with a polymer compound, whether durable or biodegradable, to form a polymer carrier system. Drug-eluting stents with such polymer carrier has a typical coating thickness of 5 μm, which is used to control the release rate of the drug to the artery wall. The same drug-polymer mix was assumed to be stored in the rhombic-shaped reservoirs of our channel stent.

#### 3.2.2. Fatigue Life Analysis

**Figure 8:**
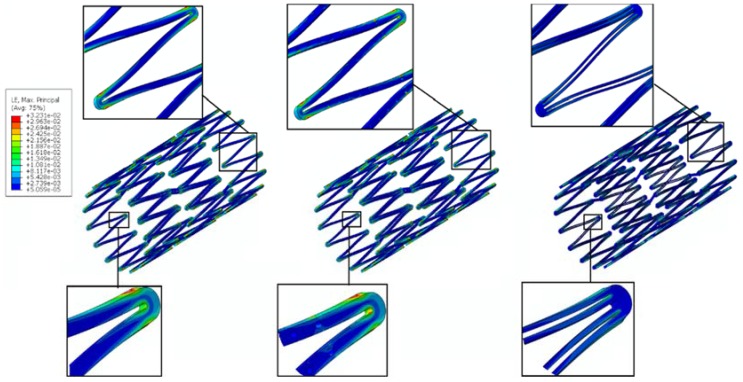
FEA contour plot comparison of strain distribution at 7.0 mm after release to a blood vessel among the conventional DES stent (**left**), conventional depot stent (**middle**), and rhombic-shaped channel stent (**right**, reservoir length 100% of the strut length).

**Figure 9:**
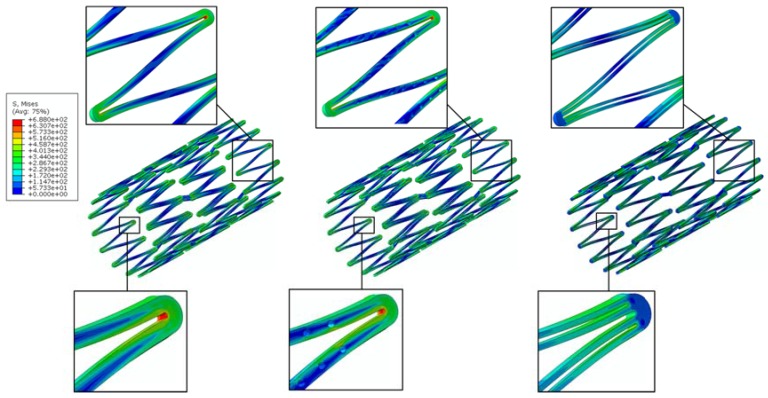
FEA contour plot comparison of von Mises stress distribution at 7.0 mm after release to a blood vessel among the conventional DES stent (**left**), conventional depot stent (**middle**), and rhombic-shaped channel stent (**right**, reservoir length 100% of the strut length).

Stent fatigue life is a major issue due to stent fractures caused by the in vivo repetitive deformations of vascular motion. For the rhombic-shaped channel stent, the reservoir length was varied from 100% to 40% of the strut length in 10% increments, while the reservoir width at the midpoint of the bar arm was maintained at 40% of the strut width for the fatigue sensitivity study. After multiple expansions and their subsequent heat treatments during manufacturing, the stent was modeled to be constrained inside a 2 mm catheter for delivery. It was then released to allow for spring-back to the target vessel diameter of 7 mm. Table 2 summarizes the FEA simulation results of the maximum strain (at 2 mm crimp and 7 mm release) and the fatigue safety factor when subjected to ±3% stent diameter oscillation, as a function of the rhombic-shaped reservoir length. For the cases of 100% and 40% of the strut length, the maximum strains at both crimp and release became lower and their fatigue safety factors jumped to about 50% higher than those of the conventional DES stent. This is amazing, considering the fact that large reservoirs were cut open on the stent struts, while the stent profile and the metal-to-artery ratio were not compromised at all. This interesting phenomenon can be attributed to the redistribution of the stresses/strains after the creation of the unique rhombic-shaped reservoirs, which effectively shifted the highly concentrated stresses/strains away from the crown regions and towards the bar arms. Figure 8 and Figure 9 show the contour plot comparison of the strain distribution and von Mises stress distribution, respectively, for the three different stent types during deployment at 7 mm. Except for the stress-concentrated regions near the crowns and reservoirs, little to no stresses/strains were found on the straight portions of the stent struts for both the conventional DES stent and the conventional depot stent, indicating that their energy distribution could be further improved. Figure 6, Figure 7, Figure 8 and Figure 9 show that the stress/strain distribution of the rhombic-shaped channel stent was spread out more uniformly than those of the other two stents, with lower intensity near the crown regions. From the energy standpoint, subjecting a higher volume of the stent structure to the same loading is a more efficient way to store energy.

**Figure 10:**
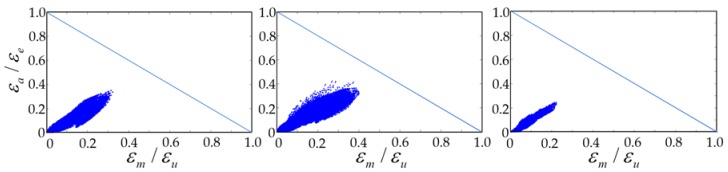
Goodman diagram comparison of conventional DES stent (**left**), conventional depot stent (**middle**), and rhombic-shaped channel stent (**right**, reservoir length 100% of the strut length).

**Figure 11:**
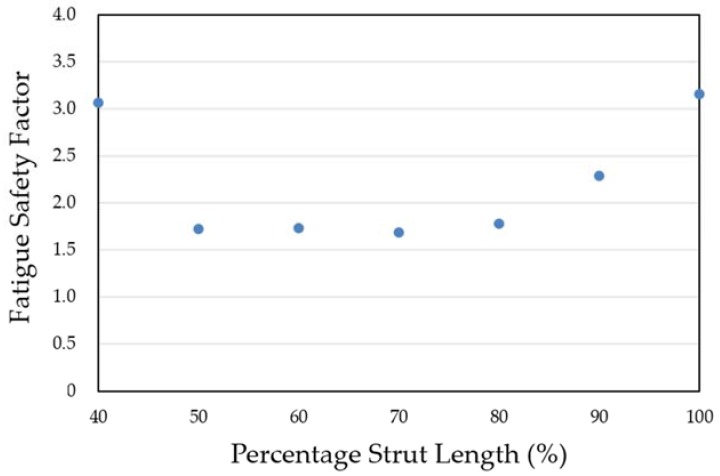
Simulation results of the fatigue safety factor as a function of the reservoir length.

Figure 10 compares the Goodman diagrams under systolic/diastolic pressure cycles for the three different stent types. All stents passed the fatigue life of 4 × 10^8^ cycles (recommended and listed in the FDA guidance documents for intravascular stents and associated delivery systems) under pulsatile fatigue loading. Calculated data were well below the Goodman diagram failure line for all cases, with the rhombic-shaped channel stent demonstrating the best stent fatigue resistance. The fatigue safety factor of this rhombic-shaped channel stent (3.16) was roughly twice that of the conventional depot stent (1.51). This is quite impressive, given the fact that both the depot stent and the channel stent had large cutouts on their stent struts. Figure 11 shows the simulation results of the fatigue safety factor as a function of variation of the reservoir length from 100% to 40% of the strut length. As shown in the figure, the fatigue safety factor decreased as the reservoir length shortened. It remained at a similar value of around 1.70 from 80% to 50% and then increased again at 40% of the strut length. This phenomenon was due to the differences in bending rigidity between the strut sections with and without reservoirs. Since the stent strut was loaded predominantly in bending, the strut section with reservoirs became more flexible, thereby inducing various shapes and degrees of deformation during expansion.

### 3.3. Channel Stent Prototyping

**Figure 12:**
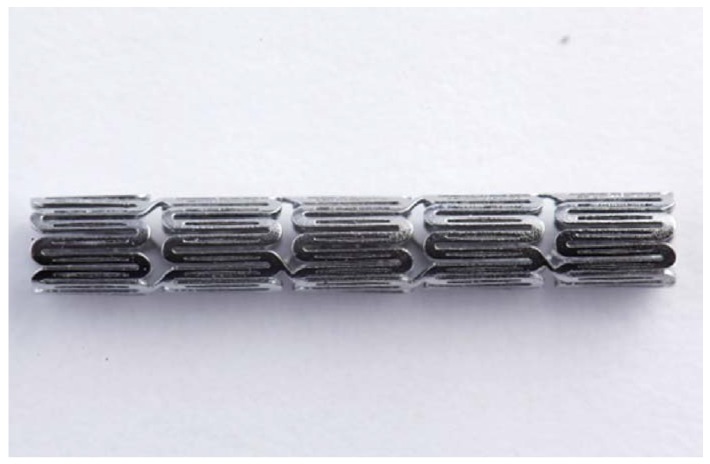
Prototype of our rhombic-shaped channel stent.

The quality of laser cutting is mainly controlled by several parameters of the laser module, including the pulse repetition rate, focal position, and assisted gas pressure. The pulse repetition rate is an important laser parameter related to the material removal and surface roughness of a stent. Typically, a higher pulse repetition rate corresponds to a better surface cutting quality. Poor cutting may cause energy to accumulate around the cutting edges and form larger heat-affected zones. The focal position, another important laser parameter, dictates the quality of the laser cutting. The focal position of our laser module was determined by measuring the kerf width on the hypotube while changing the *z*-direction distance between the laser source and hypotube. Optical microscopy was used to measure the kerf width and to observe the surface conditions. To achieve the smallest kerf width, the laser beam was focused on the hypotube surface precisely in an attempt to find the optimal focal depth. When the *z*-direction distance was between 0.27 mm and 0.51 mm, the laser beam was able to penetrate through the hypotube and cut the stent with minimal heat effects. At the *z*-direction distance of 0.37 mm, we found that the kerf width could be reduced to 23.2 μm. Figure 12 presents the rhombic-shaped channel stent for demonstration of our new design concept.

## 4. Discussion

The depot or channel stent was originally developed for the coronary indication to increase the flexibility of the drugs administered. However, we believe this new stent concept could be extended to other indications and become even more powerful. For example, as aforementioned, it could be used for potential treatment of renal artery stenosis and its associated kidney diseases simultaneously, as renal artery stenosis is reciprocally related to progressive hypertension, renal insufficiency, or kidney failure. Table 1 shows that our rhombic-shaped channel stent could increase the drug capacity to up to 7.7 times that of the conventional DES stent. Such a high drug capacity could potentially transform a stent into a drug carrier for longer-term therapies such as cancer treatment. Therefore, it is possible to design a stent that not only offers physical support to the blood vessel wall but also presents a pharmacological approach in the prevention or treatment of other diseases. The stent, acting as a drug carrier in this case, could be deployed in an artery upstream of the distal cancer site, with cancer drugs loaded in the reservoirs to be carried by the bloodstream to the downstream cancer cells for targeted therapy. Different types of drugs with different release rates could be independently arranged in each reservoir, increasing the flexibility of the drugs administered. This depot or channel stent design incorporating sequential release of multiple drugs would facilitate the management of the sequential, complex biological processes during disease treatment. The drug capacity of the rhombic-shaped channel stent could be tailored based on actual clinical needs by changing the reservoir length and width and thus the reservoir volume.

Although the drug-eluting stent has become the gold standard today for the treatment of coronary artery stenosis, its dependence of efficacy on the drug elution dose vs. elution duration remains unclear. Recent studies showed that the tissue content linearly tracked the drug elution duration in the porcine coronary artery models [52]. This implies that the persistence time of the receptor saturation and effect is more sensitive to the drug elution duration than dose, at least for Sirolimus-eluting stents such as the Cypher stent and the NEVO stent.

**Figure 13:**
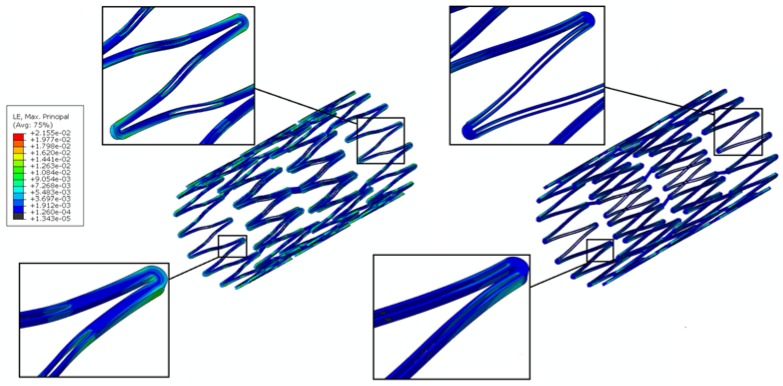
FEA contour plot comparison of strain distribution at 7.0 mm after release into a blood vessel between rhombic-shaped channel stents with reservoir lengths of 40% (**left**) and 100% (**right**) of the strut length.

The conventional depot stent has a drug capacity greater than that of the conventional DES stent; however, the creation of the circular drug reservoirs aggravated the stress concentration issue, with a 27% decrease in the fatigue safety factor relative to the convention DES stent. In contrast, the rhombic-shaped channel stent provides a smoother stress/strain transition along the bar arms and more efficient energy distribution, which in return boosts its stent fatigue resistance. As shown in this study, when the reservoir lengths were 100% and 40% of the strut length, the fatigue safety factor jumped to approximately 50% higher than that of the conventional DES stent. This is amazing, considering the large reservoir cutouts on the stent struts. Figure 13 presents a contour plot comparison of the strain distributions at 7.0 mm after release into a blood vessel between these two rhombic-shaped channel stents. For the reservoir length of 100% of the strut length, stresses/strains were drawn away from the crowns and spread to the bar arms, effectively reducing the maximum stresses/strains and enhancing the fatigue resistance. For the reservoir length of 40% of the strut length, the shorter rhombic-shaped drug reservoirs successfully drew more energy closer to the midpoints of the bar arms, as indicated by the light yellow color at the reservoir tips.

**Figure 14:**
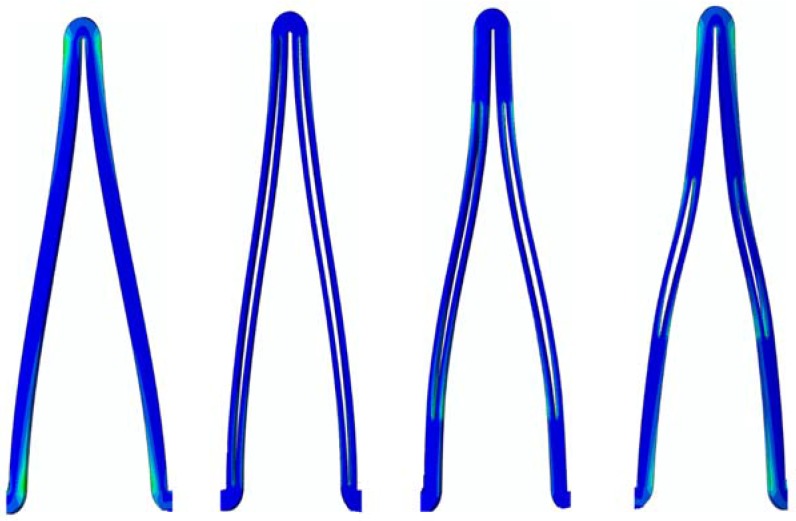
FEA comparison of stent deformed geometry at 7.0 mm after release into a blood vessel among the conventional DES stent and rhombic-shaped channel stents with reservoir lengths of 100%, 70%, and 40% of the strut length (from left to right).

However, the fatigue safety factors for the reservoir lengths of 80% to 50% of the strut length dropped to approximately 85% of that of the conventional DES stent. Figure 14 shows the comparison of stent deformed geometry at 7.0 mm among the conventional DES stent and the rhombic-shaped channel stents with reservoir lengths of 100%, 70%, and 40% of the strut length, from left to right, respectively. In the stent final deformed geometry, the originally straight bar arms were slightly bent and curved, an indication of bending moments exerted on the stent struts during stent expansion. At two hinge points, the strut shape (or curvature) changed suddenly at the quarter and three-quarter locations of the stent struts. After the creation of the rhombic-shaped reservoir, the strut was divided into two sections (non-reservoir and reservoir) with different bending rigidities. The nonreservoir section was more rigid and less flexible, while the reservoir section was the opposite. For the conventional DES stent and the rhombic-shaped channel stent with a reservoir length of 100% of the strut length, the strut bending degree and deformed geometry were similar and quite uniform along the strut length due to the more evenly distributed strut bending rigidity. For the rhombic-shaped channel stents with reservoir lengths of 80% to 50% of the strut length, their reservoir tips were closer to the hinge points, which were critical in bending control. In these cases, the stent structural integrity could be weakened and its clinical attributes compromised, including the fatigue safety factor. As a result, the stent deformed geometry looked different from the previous two cases (Figure 14), as the stent struts were clearly divided into three distinct sections with different bending rigidities. In contrast, for the rhombic-shaped channel stents with a reservoir length of 40% of the strut length, the reservoir tips were distant from the critical hinge points, and thus the fatigue safety factor was recovered.

In this study, computational simulations performed were based on the assumption that the rhombic-shaped drug reservoirs were hollow without the lateral support of the drug-polymer compound from inside, which represented the worst-case scenario in terms of the mechanical performance. Therefore, the actual fatigue safety factors of the rhombic-shaped channel stent could be even higher than those values reported in Table 2.

Having reservoirs and thus more openings on the stent struts creates more sharp edges on the stents. Such sharp edges could lead to higher risk of the restenosis or fibrosis, if proper care is not taken. Therefore, after the laser cutting and heat treatment, the stent surface has to be sand-blasted and electro-polished with the optimal combination of the voltage, current, and time to achieve a mirror-like surface finish for minimizing such risk. Table 3 summarizes the comparison of the strut/reservoir edge length among the three different stent types: the conventional DES stent, the conventional depot stent, and our rhombic-shaped channel stent with various strut lengths. The rhombic-shaped channel stent creates more edges which need to be smoothed out during manufacturing to ensure its success.

## 5. Conclusions

A rhombic-shaped channel stent with enhanced drug capacity and stent fatigue life was investigated. The design concept was to shift the highly concentrated stresses/strains away from the stent crown regions, allowing the stress-free bar arms to carry more loadings by cutting the rhombic-shaped drug reservoirs on the bar arms. The total drug capacity was thus several times greater, and the fatigue safety factor was 50% higher, than those of the conventional DES stent. Therefore, our rhombic-shaped channel stent can not only carry larger drug loadings but also has an enhanced fatigue safety factor at the same time, a combination of features exceeding those of the current DES stent. A prototype of the rhombic-shaped channel stent was manufactured for the demonstration of our stent design concept.

This design concept could potentially transform a stent into a drug carrier for longer-term therapies such as cancer treatment. We could design a stent to be positioned in an artery upstream of the cancer site to release cancer drugs to be carried to the distal cancer cells via the bloodstream for programmable and targeted therapy. The findings of this paper provide an excellent example of improvement of the clinical performance of the depot or channel stent without compromising other important clinical attributes.

## Figures and Tables

**Table 1:** Comparison of total drug capacity.

Stent Type	Total Drug Capacity
Conventional DES stent	100%
Conventional depot stent	165%
Rhombic-shaped channel stent (100% SL)	766%
Rhombic-shaped channel stent (90% SL)	696%
Rhombic-shaped channel stent (80% SL)	619%
Rhombic-shaped channel stent (70% SL)	541%
Rhombic-shaped channel stent (60% SL)	463%
Rhombic-shaped channel stent (50% SL)	386%
Rhombic-shaped channel stent (40% SL)	307%

**Table 2:** Maximum strain and fatigue safety factor as a function of narrowed strut width.

Stent Type	*ε*_max_, Crimped to 2 mm	*ε*_max_, Released to 7 mm	Fatigue Safety Factor (*FSF*)
Conventional DES stent	6.09%	2.52%	2.06 (100%)
Conventional depot stent	6.66%	3.23%	1.51 (73%)
Rhombic-shaped channel stent (100% SL)	4.54%	1.99%	3.16 (154%)
Rhombic-shaped channel stent (90% SL)	5.75%	2.49%	2.29 (111%)
Rhombic-shaped channel stent (80% SL)	7.28%	3.14%	1.78 (87%)
Rhombic-shaped channel stent (70% SL)	6.33%	2.71%	1.69 (82%)
Rhombic-shaped channel stent (60% SL)	7.57%	3.33%	1.73 (84%)
Rhombic-shaped channel stent (50% SL)	7.49%	3.29%	1.72 (84%)
Rhombic-shaped channel stent (40% SL)	5.61%	2.40%	3.07 (149%)

**Table 3:** Comparison of strut/reservoir edge length.

Stent Type	Strut/Reservoir Edge Length (mm)
Conventional DES stent	1450 (100%)
Conventional depot stent	1800 (124%)
Rhombic-shaped channel stent (100% SL)	2772 (191%)
Rhombic-shaped channel stent (90% SL)	2652 (183%)
Rhombic-shaped channel stent (80% SL)	2519 (174%)
Rhombic-shaped channel stent (70% SL)	2385 (165%)
Rhombic-shaped channel stent (60% SL)	2252 (155%)
Rhombic-shaped channel stent (50% SL)	2119 (146%)
Rhombic-shaped channel stent (40% SL)	1986 (137%)
